# A structured model for immune exposures

**DOI:** 10.1093/database/baaa016

**Published:** 2020-04-11

**Authors:** Randi Vita, James A Overton, Patrick Dunn, Kei-Hoi Cheung, Steven H Kleinstein, Alessandro Sette, Bjoern Peters

**Affiliations:** 1 Division for Vaccine Discovery, 9420 Athena Circle La Jolla Institute for Allergy and Immunology, La Jolla, CA 92037, USA; 2 Knocean Inc., 2 - 107 Quebec Ave Toronto M6P 2T3, Ontario, Canada; 3 ImmPort Curation Team, NG Health Solutions, 2101 Gaither Road Rockville, MD 20850, USA; 4 464 Congress Ave Department of Emergency Medicine, Yale University, New Haven, CT, 06519 USA; 5 Interdepartmental Program in Computational Biology and Bioinformatics, Yale University, 464 Congress Ave New Haven, CT, 06519 USA; 6 Department of Pathology, Yale School of Medicine, 464 Congress Ave New Haven, CT, 06519 USA; 7 Department of Medicine, University of California San Diego, 9500 Gilman Dr La Jolla, CA, 92093 USA

## Abstract

An Immune Exposure is the process by which components of the immune system first encounter a potential trigger. The ability to describe consistently the details of the Immune Exposure process was needed for data resources responsible for housing scientific data related to the immune response. This need was met through the development of a structured model for Immune Exposures. This model was created during curation of the immunology literature, resulting in a robust model capable of meeting the requirements of such data. We present this model with the hope that overlapping projects will adopt and or contribute to this work.

## Introduction

The extremely diverse antibody and T cell repertoire exists as a result of early VDJ (variable (V), diversity (D) and joining (J)) recombination events, creating an enormous possibility of different sequences (1). Any given antibody or T cell may protect from an infection or may never serve a purpose. Which specific antibodies or T cells are called to action depends upon the specific immune exposures that are encountered throughout one’s lifetime. Immune exposures are those exposures to foreign or self-entities capable of triggering an adaptive immune response and include such events as vaccinations, infections and environmental exposures to anything capable of causing a disease. In the course of describing immunological data in a structured manner, to be housed in the Immune Epitope Database (IEDB) (2), it became necessary to formulate an ontology driven, standardized way of capturing and communicating the components of an immune exposure event, as no previous method could be identified. Here, we will describe the main components of this model and how its implementation to experimental data has improved public immunology resources.

The immune exposure represents the beginning of the adaptive immune response. The antibodies or T cells specific to any particular threat exist in small numbers until their relevant immune exposure event occurs. For example, the number of antibodies capable of recognizing *Plasmodium falciparum* will not increase until one contracts malaria, at which time they will increase as part of a protective immune response. Thus, levels of *P. falciparum* antibodies are measurable in individuals who have or recently had malaria, while they are not usually measurable in healthy donors, who have never contracted malaria. Therefore, it is critical to know the immune exposure history of donors being studied in immunological assays in order to appropriately interpret the outcomes of these studies.

## Process and results

The IEDB was the original driving force for the need of an immune exposure model. The IEDB was established by a National Institute of Allergy and Infectious Disease contract in 2004 as a public resource to house experimental data demonstrating an epitope specific adaptive immune response (3). This data is primarily entered by manual curation from the scientific literature on an ongoing basis, including greater than 95% of all published data. The IEDB content is updated weekly. How the IEDB represents the details of published experiments in a searchable, structured format to facilitate advances in the field of immunology is described fully in an earlier publication (2). To provide this structure, we analyzed the scenarios being studied within the immunology literature and broke out the individual components and processes of the adaptive immune exposure process. This resulted in a standardized model with four main aspects; Exposure Process, Exposure Material, Disease Name and Disease Stage.

Firstly, there is the process of how the subject became exposed to the specific material that the antibody or T cell response recognizes, the Exposure Process. This process may be an administration, in the case of vaccinated subjects. There may be a disease process that occurred, whereby the subject became infected with a pathogen. Another common process is the documented exposure to something in the environment capable of causing a disease, but for which no disease occurred, for example when a subject lives with a known tuberculosis-infected subject, but remains healthy despite this constant exposure. Also, during a transplantation procedure, one may become exposed for the first time to foreign antigens present on the donor tissue.

Secondly, there is what the subject was exposed to, the Exposure Material. Exposure materials may include the vaccine that was administered, the pathogen or allergen triggering a disease or any relevant entity to which the subject was exposed. In addition to the exposure process and material, there is also the outcome of this exposure to be considered. Did the subject become diseased as a result? Lastly, if a disease occurred, it is also necessary to describe the stage of the disease at the time of the experiment. The immune response will differ in a subject in the acute stage of an infection compared to a subject who had the same infection many years in the past.

This model was initially applied to the IEDB using ‘in house’ terms rather than formal ontology terms. This was a result of timing and evolving knowledge. At the onset, not all needed terms were available in existing ontologies, and equally important was the need to collect more data to ensure the model was robust. To date, the IEDB has data accumulated from more than 20 000 scientific articles, amounting to more than 2 million manually curated immunological experiments. Throughout the process of accumulating more data and more experience stewarding such data, the IEDB’s implementation of the immune exposure model gradually moved to structured ontology terms with logical relationships. A file (immune_exposure.owl) containing the ontology terms we used for this model can be downloaded at: https://github.com/jamesaoverton/immune-exposure-validation. This process was driven by the desire to standardize and organize our data to improve our user experience but also to make our large dataset more interoperable and reusable by other projects. The vast amount of IEDB data was used to test the validity of the model and to determine which ontology terms were needed to represent the state of immunological experimentation. This has resulted in contributions to many ontology efforts, as described below. For each ontology term utilized by the IEDB, we store the term Internationalized Resource Identifier (IRI) and perform weekly updates to ensure those terms still exist within each source ontology.

The Exposure Process types encountered in the literature grew over time and fell into three main categories: Administrations, Occurrences of disease and Exposures without disease, shown in Table 1. The Administration process type is used to describe scenarios where the immune system is first exposed via a direct, controlled process such as a vaccination event. The Occurrence of the disease process is used to describe situations where the immune system becomes exposed during a disease process, for example, a viral infection. The Exposure process type is used to depict environmental events whereby the immune system is exposed, but this exposure event does not result in a disease process. Administration and Occurrences of disease processes are represented by the Ontology for Biomedical Investigations (OBI) (4). Exposure processes were initially also modeled using OBI, but can recently be modeled using the Environmental Conditions, Treatments and exposures Ontology (ECTO) (5), to which we are currently transitioning. The IEDB controls how this data is collected via a controlled vocabulary drop down list that grew as new types were encountered.

**Table 1:** Exposure process types

**Exposure process category**	**Exposure process type**
Administration	Vaccination
Administration	Infectious challenge
Administration	Transplantation
Occurrence of disease	Infectious disease
Occurrence of disease	Allergic disease
Occurrence of disease	Autoimmune disease
Occurrence of disease	Cancer
Exposure without disease	Asymptomatic infection
Exposure without disease	Exposure with immune reactivity
Exposure without disease	Documented exposure
Exposure without disease	Exposure to ubiquitous agent

The Exposure Materials are made up of a wide variety of material entities that can be described by several different resources. The Vaccine Ontology (6) can be used to model vaccines and NCBI Taxonomy (7) describes exposures to organisms, such as infection with viruses, bacteria or parasites. Other ontologies, as applicable, are used for different entity types that elicit immune responses, for example Chemical Entities of Biological Interest (ChEBI) (8) is used for exposures to chemicals, drugs and small molecules. These diverse terms are entered into the IEDB via a Finder web application, custom built to allow curators access to terms from multiple ontologies.

The Disease Name and Stage were originally described using an accumulated list of disease terms encountered in the literature over many years, with a focus on infectious, allergic, autoimmune and transplant-related diseases due to the scope of the IEDB project. The list of disease terms was eventually mapped to terms in the Disease Ontology (DO) (9), which is also now used to organize how disease terms are entered into the IEDB. Any terms needed that were not already present were requested as new terms to fill the gap in the mapping process. Disease Stages found in the IEDB relevant immunology literature amounted to just a few terms, including acute, chronic and post. These terms were requested by the IEDB and created in the Ontology for General Medical Science (OGMS) (10). Restriction to this set of standardized terms is facilitated by a drop-down list. If any additional disease stage terms are needed in the future, those requests may also be made to OGMS.

After confidence in this model was established by its ability to consistently model the data in the IEDB over many years, it was extended to other immunology resources, including the Immunology Database and Analysis Portal (ImmPort) (11). ImmPort is an extensive data warehouse containing experimental data and metadata related to human and model organism immunology studies. ImmPort receives direct submissions of raw data and protocols from clinical trials, mechanistic studies and novel method developments from immunologists. Similar to the IEDB, ImmPort utilizes structured data fields and free-text fields describing many details of immunological experiments. Details regarding the structure and accessibility of ImmPort can be found in an earlier publication (11). ImmPort captures many details of study subjects and experimental protocols in a variety of spreadsheet templates. Previously, the information regarding the host’s immune exposure was entered via several free text fields by the researchers when submitting their data. The Immune Exposures Model was first applied to ImmPort data derived specifically from The Human Immunology Project Consortium (HIPC) (12) studies. The motivation for this approach was the strong interest in data standards and the facilitation of interoperability by the consortium. The existing HIPC data in ImmPort was manually analyzed and mapped to ontology terms following the IEDB model (13). ImmPort opted to store ontology term names that are verified via lookup tables that are maintained via a periodic review of the ontology sources. Examples of mapped immune exposure data from the IEDB and ImmPort are shown in Table 2. Additionally, and significantly, the data entry process in ImmPort was updated to adopt the immune exposure model to enhance the standardization of annotation going forward. This required changes to data upload templates, including the addition of new fields and directing data providers to the preferred terms to enter for each field, using drop-down lists. Updates to the data structure were required to store the four main components of the model (Exposure Process, Exposure Material, Disease Name and Disease Stage) as separate concepts verses a free text field.

**Table 2:** Example modeled immune exposure data

**Source**	**Captured scenario**	**Exposure process**	**Exposure material**	**Disease name**	**Disease stage**
IEDB	Penicillin allergic subjects	Allergic disease	Penicillin	Penicillin allergy	Chronic
IEDB	Healthy donors living in a malaria endemic area	Exposure to ubiquitous agent	Plasmodium falciparum		
ImmPort	Blood bank donations identified as sero-positive for antibodies against dengue serotype 2	Exposure with immune reactivity	Dengue virus 2		
ImmPort	Subjects with severe West Nile fever	Infectious disease	West Nile virus	West Nile fever	Acute

## Discussion

One of the main advantages of standardizing data via ontology terms is the ability to apply reasoning to the captured data. Incorporation of the Immune Exposures Model facilitates validation across multiple data fields, as shown in Table 3. The values entered in one field of the data resource can be used to validate what is entered into another field. For example, if the Exposure Process is ‘Infectious Disease’, then a pathogen must be entered into the Exposure Material field and an infectious disease should be entered into the Disease Name. If any value is entered into the Disease Name, a value should also be entered into the Disease Stage Field. Likewise, if no value is entered into the Disease Name, no value should be entered into the Disease Stage Field. This type of inter-field validation was incorporated into both the IEDB and ImmPort. It is used to both prevent erroneous data entry and identify existing errors. Furthermore, validation that limits entered values to terms from the specified ontologies makes data entry easier and reduces data entry errors.

**Table 3:** Requirements of the immune exposure model

**Exposure process type**	**Exposure material**	**Disease name**	**Disease stage**
Vaccination	Required	None	None
Infectious challenge	Required	Optional	Optional
Transplantation	Required	None	None
Infectious disease	Required	Required	Required
Allergic disease	Required	Required	Required
Autoimmune disease	None	Required	Required
Cancer	None	Required	Required
Asymptomatic infection	Required	None	None
Exposure with immune reactivity	Required	None	None
Documented exposure	Required	None	None
Exposure to ubiquitous agent	Required	None	None

The limitations of our model are mainly due to the availability of appropriate ontology terms. We were able to overcome this challenge by making new term requests as needed and becoming actively involved in ontology development when necessary, as previously described (14). However, it must be noted that many of the terms we are currently using may be considered high level. For the IEDB’s current application, these terms are sufficient; nevertheless, if a new project wishes to adopt this immune exposure model and requires more specific terms for their needs, new ontology terms can be requested at any time. This is the benefit of selecting ontologies with active developer and user communities. Additionally, as part of the IEDB’s routine maintenance, we perform annual reviews of ontology term use, with the goal of identifying overloaded terms that may require further specification. In those cases, we make new term requests and map our existing data to new, more specific terms.

We intend to continue to improve and extend the benefits of this model. As mentioned above, continued evaluation of the ontology terms used may result in improved detail of the collected data. The validation measures within the IEDB continue to enforce adherence as new data is added. The similar validation retroactively applied to ImmPort data is currently being used to identify errors in existing data, which will be soon presented publicly as an example of the utility of this model. Such structured data can also be exploited to enable cross resource queries. Because multiple resources, including the IEDB and ImmPort, now utilize the same Immune exposures model, queries across the multiple datasets is now facilitated. We demonstrated proof of this concept by loading data from ImmPort, IEDB and EuPathDB (15) into a preliminary RDF triple store, enabling queries across all three data resources, for example retrieving all data where hosts were exposed to the same pathogen, regardless of disease state and including vaccinated subjects that were never infected. We will further this project as more data fields are included in the triple store and as EuPathDB hopes to adopt the immune exposures model in the near future. We will also continue to solicit additional use cases whose data can be similarly cross-queried.

The utility and strength of this Immune Exposure model is proven through its application to more than two million published experiments and its adoption by multiple public resources. The Immune Exposure model is also now being implemented by additional HIPC projects outside of ImmPort, including the HIPC Signatures knowledgebase and data re-analysis project. Standardization of data allows better reuse and interoperability, as previously isolated data sets can now easily be combined in meaningful ways. We present our model here with the hopes that other projects housing similar datasets will consider structuring their data using this model. We welcome collaboration and feedback.
